# Human p53 interacts with the elongating RNAPII complex and is required for the release of actinomycin D induced transcription blockage

**DOI:** 10.1038/srep40960

**Published:** 2017-01-19

**Authors:** Barbara N. Borsos, Ildikó Huliák, Hajnalka Majoros, Zsuzsanna Ujfaludi, Ákos Gyenis, Peter Pukler, Imre M. Boros, Tibor Pankotai

**Affiliations:** 1 Department of Biochemistry and Molecular Biology, University of Szeged, Szeged, 6726, Hungary; 2 Department of Molecular Genetics, Erasmus University Medical Centre, Rotterdam, PO Box 2040, 3000 CA, The Netherlands; 3 Institute of Biochemistry, Biological Research Centre, Szeged, 6726, Hungary

## Abstract

The p53 tumour suppressor regulates the transcription initiation of selected genes by binding to specific DNA sequences at their promoters. Here we report a novel role of p53 in transcription elongation in human cells. Our data demonstrate that upon transcription elongation blockage, p53 is associated with genes that have not been reported as its direct targets. p53 could be co-immunoprecipitated with active forms of DNA-directed RNA polymerase II subunit 1 (RPB1), highlighting its association with the elongating RNA polymerase II. During a normal transcription cycle, p53 and RPB1 are localised at distinct regions of selected non-canonical p53 target genes and this pattern of localisation was changed upon blockage of transcription elongation. Additionally, transcription elongation blockage induced the proteasomal degradation of RPB1. Our results reveal a novel role of p53 in human cells during transcription elongation blockage that may facilitate the removal of RNA polymerase II from DNA.

The transcription of protein-coding genes in eukaryotic cells is catalysed by a 12-subunit (RPB 1–12) enzyme, RNA polymerase II (RNAPII). In the process of initiation, the general transcription factors (TFIIB, D, E, F and H) are recruited to promoters and the unphosphorylated RNAPII associates with them, forming the pre-initiation complex (PIC)^1^. At this stage, the C-terminal domain (CTD) of RPB1 becomes phosphorylated at its Ser-5 residues (S5P) by Cdk-7, while during a later phase of transcription elongation, RPB1 CTD will be phosphorylated at its Ser-2 residues (S2P) by Cdk-9^2^. These modifications are tightly associated with RNA capping, splicing and polyadenylation^3^,^4^.

Additionally, DNA damage-induced transcription blockage also affects the phosphorylation state of RPB1. This could activate the binding of specific factors to the RNAPII complex with various outcomes^5^. If irreversible transcription blockage occurs, the RNAPII is polyubiquitylated and degraded by the 26S proteasome^6^,^7^. The ubiquitin-proteasome system (UPS)-mediated elimination of RNAPII from the site of DNA damage allows the repair of DNA lesions^8^,^9^,^10^,^11^. It has been shown that proteins in different DNA damage repair processes, such as CSA (Cockayne syndrome A), BRCA1 (Breast cancer 1), BARD1 complex (BRCA1-associated RING) and NEDD4 (neural precursor cell expressed, developmentally down-regulated 4), are involved in the ubiquitylation of RNAPII^12^,^13^,^14^,^15^,^16^,^17^. Recently it has also been reported that the 26S proteasome can be directly recruited to the damaged chromatin region for the efficient degradation of the ubiquitylated RNAPII^18^. In accordance with this, it has been demonstrated that the S2P forms of RPB1 are better substrates for proteasomal degradation, which indicates that the elongating form of RNAPII can be targeted for degradation^19^. Finally, the proteasome itself is also present at transcriptionally active gene regions where RNAPII density is high, which underlines the notion that the proteasome might have a role at chromatin sites, where transcription arrest occurs^20^,^21^.

p53 is a stress-activated, sequence-specific transcription factor, which binds to consensus sequences at the promoter and distal regions of its target genes (e.g. *Bax, Bad*, and *P21*). p53 also acts as a tumor suppressor that can induce apoptosis upon serious DNA damage by releasing pro-apoptotic factors from the mitochondria or activating the transcription of pro-apoptotic factors, and it can also induce cell cycle arrest^22^,^23^.

Upon DNA damage, transcription blockage occurs, p53 is phosphorylated by ATR (ataxia telangiectasia and Rad3 related) kinase, and DNA repair proteins are activated to remove the lesions^5^. Additionally, upon transcription elongation blockage, ATM-mediated Ser-15 phosphorylation and p300-mediated Lys-382 acetylation stabilises p53^5^,^24^,^25^. These two post-translational modifications of p53 have an indispensable role in p53-mediated signalisation during cell cycle arrest and apoptosis induction^26^,^27^,^28^.

In addition to its role as a sequence-specific DNA-binding factor in transcription initiation, p53 could facilitate transcription by interacting with proteins involved in transcription elongation (e.g. TFIIH, ELL and hPAF1C)^29^,^30^,^31^. In accordance with this, the human p53 protein has been reported to associate with coding regions of RNAPII-transcribed genes in a heterologous system^32^. It was previously shown that Dmp53 (*Drosophila melanogaster* p53) was localised at transcriptionally active regions on the *Drosophila* polytene chromosomes and that the phosphorylation state of RPB1 CTD influenced its localisation^33^.

Here we describe the results of experiments investigating whether the p53 protein was able to bind to gene regions that were not its direct targets, due to the presence of specific binding site(s) at their regulatory regions. We examined whether the binding of p53 to non-canonical target gene regions changed upon transcription elongation blockage induced with a high dose of actinomycin D (ActD). Furthermore, we asked whether an interaction between p53 and phosphorylated forms of RPB1 (S2P and S5P) could be detected in human cells and how it was altered upon ActD-induced inhibition of transcription elongation. We found that p53 binds to gene regions that are non-canonical targets and the binding takes place presumably through the interaction between p53 and RPB1. During transcription elongation blockage, RPB1 occupancy is reduced on the chromatin, while p53 binding can be detected at most of the examined promoters and distal gene bodies, particularly shortly after the induction of the transcription blockage. Upon transcription stress, the cellular p53 level is increased, while the RPB1 protein level is decreased as a result of proteasomal degradation of RPB1.

These observations highlight a mechanism by which transcription blockage that occurs due to different types of DNA damage could be resolved. The mechanism we propose here helps to understand how the RNAPII is degraded on a damaged transcribed unit in order to allow access for repair proteins. We assume that by this mechanism, cells can avoid producing truncated or mutated transcripts from essential genes that would endanger cell viability.

## Results

### Human p53 interacts and co-localises with RNAPII on chromatin

**Figure 1:**
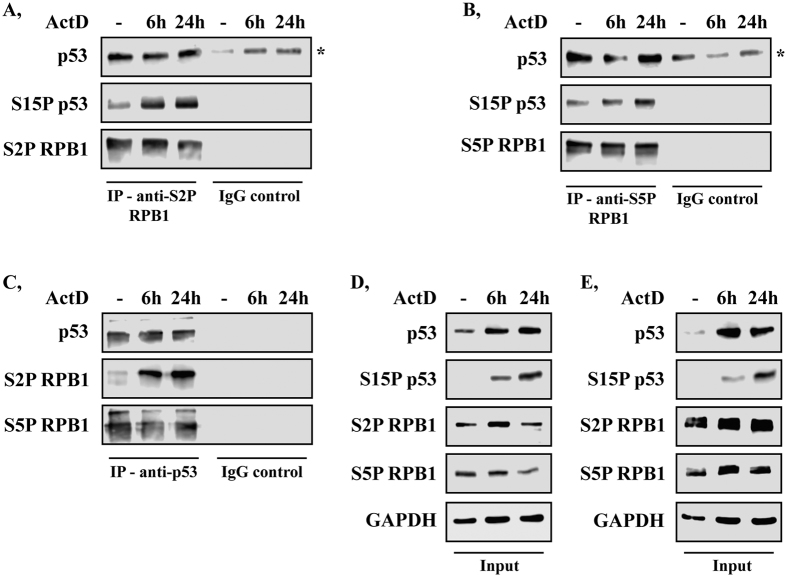
Human p53 interacts with RPB1 both under normal circumstances and upon ActD treatment. (**A**,**B**) The transcriptionally active forms of RPB1 (S2P and S5P) were immunoprecipitated and the RPB1-p53 interaction was validated by detection of p53 and S15P p53 with immunoblot using specific antibodies. (**C**) Asterisks show the unspecific signal of the IgG heavy chain. The reversal immunoprecipitation was performed by a p53-specific antibody. The p53-S2P RPB1 and p53-S5P RPB1 interactions were detected by specific antibodies against S2P and S5P RPB1 while IgG controls were used as the negative controls. (**D**,**E**) Western blots were performed on the input protein samples to prove the equal protein quantity for each sample used for the S2P S5P RPB1 (**D**) and p53 (**E**) immunoprecipitations. GAPDH was used to show the equal loading of the samples.

p53 has been reported to interact with proteins involved in transcription elongation, such as TFIIH (Transcription Factor II H)^30^, ELL (Elongation factor RNA polymerase II)^31^ and hPAF1C (RNA polymerase-associated factor 1 complex)^34^, which suggests that p53 may have a role during transcription elongation. Interaction between human p53 and RPB1 has been shown in yeast^32^, however, the interaction between the two proteins in human cells in relation to transcription elongation has not been investigated yet. In order to study whether interaction between human p53 and RPB1 is detectable in U2OS cells, we performed co-immunoprecipitation (co-IP) experiments using specific antibodies that recognise Ser2-phosphorylated RPB1 (S2P RPB1) or Ser5-phosphorylated RPB1 (S5P RPB1) forms (Fig. 1A,B). We performed the co-IP experiments both under normal conditions and after 6 and 24 h of transcription blockage induced by actinomycin D (ActD). We found that p53 was co-immunoprecipitated with S2P and S5P forms of RPB1 (lane 1 of Fig. 1A,B, upper panel, respectively). To prove that RPB1 interacts with S15-phosphorylated p53 (S15P p53), which is the active form of p53, we performed co-IP experiments in control and ActD-treated cells. We found that the S15P p53 interacts with S2P and S5P RPB1 (lane 1 of Fig. 1A,B, middle panel, respectively). These results indicate that p53 interacts both with the initiating (S5P) and elongating (S2P) froms of RPB1 under normal conditions. To reveal whether the interaction between the two proteins was mediated by DNA, we used MNase before immunoprecipitation in order to eliminate DNA. We found that under normal conditions, the removal of DNA did not change the interaction between p53 and the elongating S2P RPB1 (Supplementary Figure 1A, lane 4).

To examine whether p53 also interacts with S2P and S5P forms of RPB1 during transcription blockage induced by a high concentration of ActD, we performed further co-immunoprecipitation experiments on samples obtained 6 and 24 h after ActD treatment. p53 could be co-immunoprecipitated with the phosphorylated forms of RPB1 at both time points after ActD treatment (lane 2–3 of Fig. 1A,B, upper panel, respectively). Similarly, we found that the S15-phosphorylated p53 also interacted with S2P and S5P RPB1 (lane 2–3 of Fig. 1A,B, middle panel, respectively). Additionally, the interaction of S2P RPB1 and p53 in MNase-treated samples could be detected both after 6 and 24 h of ActD treatment (Supplementary Figure 1A, lane 5–6).

To verify the above results on p53 and RNAPII interaction, we performed reciprocal co-IP experiments by immunoprecipitating with a p53-specific antibody and detecting specific RPB1 forms by Western blot analysis in the precipitated material (Fig. 1C). The results strengthened our previous experimental data that the two proteins interact with each other.

Taken together, the results of the co-immunoprecipitation experiments demonstrated that, both under normal conditions and upon transcription elongation blockage, p53 and S15P p53 interacted with the S2P form of RPB1 (Fig. 1A–C). This could imply that p53 has a role in transcription elongation during transcription-coupled stress conditions in human cells.

**Figure 2:**
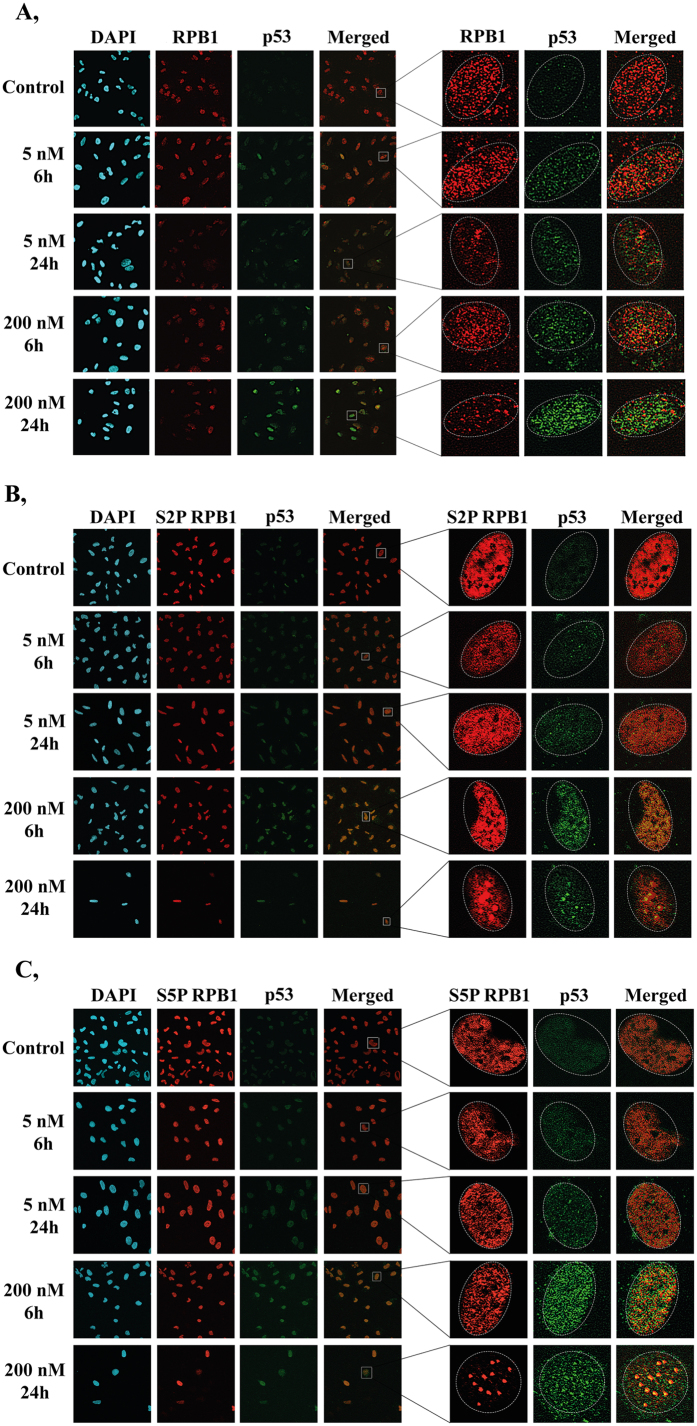
p53 and RPB1 co-localise to discrete nuclear foci in U2OS cells upon ActD-induced transcription elongation blockage. (**A**–**C**) Co-immunostaining with p53 (green) and RPB1 (red), S2P RPB1 (red) or S5P RPB1 (red), respectively. Only chromatin-bound proteins are detected, because nuclear soluble proteins were eliminated during the immunostaining process (see Methods). Immunostainings were performed both under normal conditions and after 6 and 24 h treatments of 5 and 200 nM doses of ActD. Staining with DAPI (blue) was used to visualise nuclei.

To further support these results, we performed immunostainings on U2OS cells to detect the localisation of p53 and RPB1 protein forms upon treatment with a low (5 nM) or high (200 nM) concentration of ActD. In order to detect only the chromatin-bound proteins, we eliminated the soluble nuclear proteins before performing the immunostaining experiments. Upon ActD treatment, we detected higher levels of chromatin-bound p53 and lower levels of RPB1 compared to the untreated controls (Fig. 2A). Our results show that p53 co-localises with the S2P and S5P forms of RPB1 when treated with a high concentration of ActD (Fig. 2B,C), which is in accordance with the co-IP results shown on Fig. 1.

Interestingly, 24 h after treatment with a high concentration of ActD, S2P and S5P RPB1 co-localise with p53 at discrete nuclear foci, suggesting that strong RPB1-p53 interactions might take place at locations where DNA damage-induced transcription blockages occur (Fig. 2B,C).

### ActD treatment results in DNA double-strand breaks leading to transcriptional silencing

**Figure 3:**
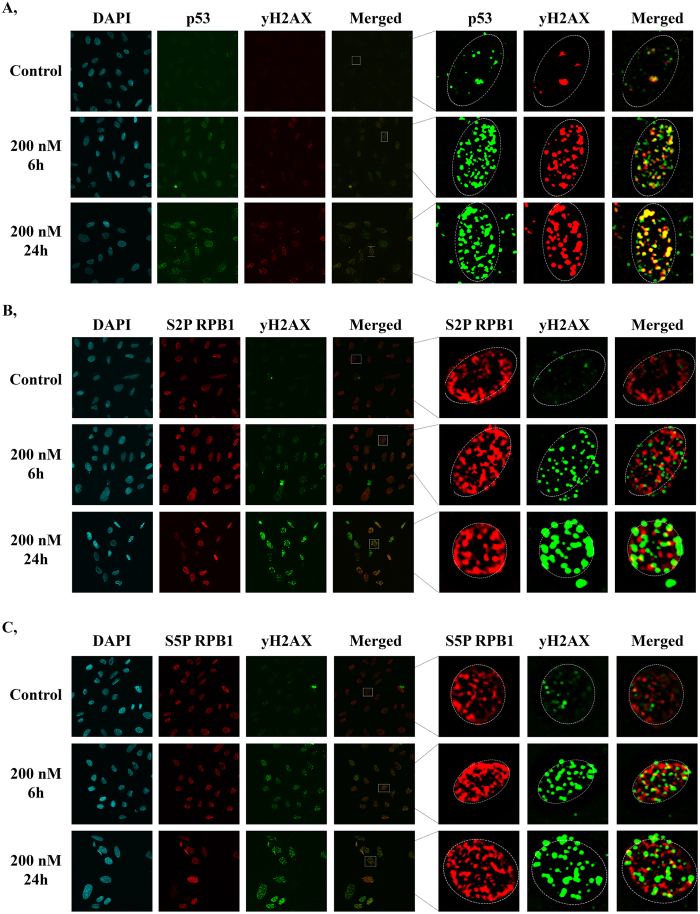
p53 and yH2AX co-localise at discrete nuclear foci upon transcription elongation blockage. (**A**) Co-immunostaining with yH2AX (red) and p53 (green). (**B**,**C**) Co-immunostaining with yH2AX (green) and S2P RPB1 (red) or S5P RPB1 (red), respectively. Only chromatin-bound proteins were detected, because nuclear soluble proteins were eliminated during the immunostaining process (see Materials and methods). Immunostainings were performed both under normal conditions and after 6 and 24 h treatments of 200 nM ActD. Staining with DAPI (blue) was used to visualise nuclei.

It has been reported recently that ActD treatment results in DNA double-strand breaks^35^. In addition, in response to DNA damage, DNA-PK- (DNA-dependent protein kinase) and ATM-(Ataxia telangiectasia mutated) mediated local inhibition of transcription occurs at the site of the damage, leading to the ubiquitin-mediated degradation of RNAPII^7^,^9^,^10^. In order to demonstrate that, in our experimental setup, the ActD-induced transcription blockage resulted in DNA double-strand breaks (DSBs), we examined the distribution of γH2AX and p53 by immunostaining, 6 and 24 h after ActD treatment (Fig. 3). While in control cells, low levels of γH2AX and p53 could be observed, both γH2AX and p53 foci numbers increased 6 and 24 h after ActD treatment (Fig. 3A), highlighting the locations of DSBs. Additionally, after 6 and 24 h ActD treatment, γH2AX and p53 co-localise in discrete nuclear foci, marking the sites where active DNA damage repair takes place (Fig. 3A, middle and lower panel).

To reveal whether the ActD-induced DNA damage leads to transcriptional silencing, we performed immunostainings and tested the co-localisation frequency between γH2AX and the phosphorylated forms of RPB1 following 6 and 24 h of ActD treatment. We found that neither γH2AX and S2P RPB1 nor γH2AX and S5P RPB1 co-localise with each other (Fig. 3B,C), strengthening the hypothesis that transcription is inhibited where DNA repair takes place.

We concluded that the DNA damage response may result in complete transcriptional silencing at the DNA break sites. Since ATM and DNA-PK could phosphorylate the serine-15 residue of p53 in response to DNA damage, we believe that RNAPII degradation is mediated by p53^36^,^37^,^38^,^39^. This mechanism is likely to be activated by the stalling of RNAPII and further promoted by the DNA damage-induced PI3K kinases.

### p53 protein is stabilised, while RPB1 is degraded upon ActD treatment

**Figure 4:**
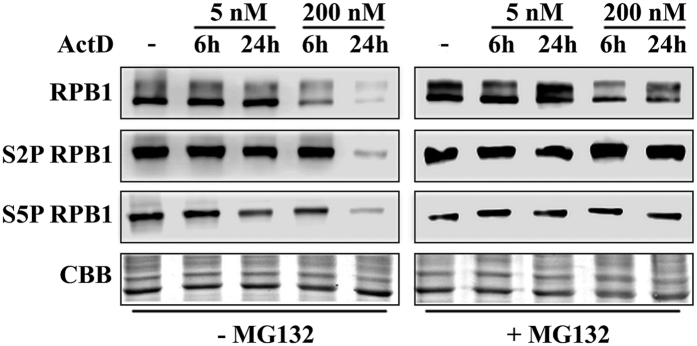
ActD treatment destabilises RPB1 through proteasome-mediated degradation. Western blot detection of RPB1, S5P RPB1 and S2P RPB1 proteins in U2OS cells both under normal conditions and when treated with 5 and 200 nM ActD. Each experiment was performed in the absence (left panel) or in the presence of 20 μM MG132 proteasome inhibitor (right panel). Coomassie Brilliant Blue staining was used to show the equal loading of the samples.

To further investigate the changes in the levels of p53 and RPB1 during transcription elongation blockage, we treated U2OS cells for 6 or 24 h with low (5 nM) or high (200 nM) concentrations of ActD then we examined p53 and RPB1 protein levels in whole cell extracts. The total p53 level increased shortly after treatments of both low and high concentrations of ActD (Supplementary Figure 2). Conversely, we detected Ser15-phosphorylated p53 (S15P p53) only upon 200 nM ActD treatment, which supports the hypothesis that this post-translationally modified form of p53 is present only following transcription elongation blockage or DNA double-strand breaks^5^ (Supplementary Figure 2). On the other hand, RPB1, as well as S5- and S2-phosphorylated RPB1, levels were reduced particularly after 24 h of high concentration ActD treatment (Fig. 4, left panel). These results indicate that p53 is stabilised, while RPB1 may be degraded upon transcription elongation blockage.

It has already been demonstrated that transcription blockage can lead to ubiquitin-mediated proteasomal degradation of RNAPII^7^,^21^. To reveal whether the reduction in the level of RPB1 protein was a consequence of its proteasomal degradation, we treated the cells with MG132 proteasome inhibitor (20 μM). We found that the levels of both RPB1 and its transcriptionally active forms (S5P and S2P) remain mostly unchanged upon ActD treatment (Fig. 4, right panel; Supplementary Figure 2). These results support the hypothesis that during exposure to stress, RPB1 is degraded by the 26S proteasome.

### p53 binds to gene regions that are not its direct targets and its binding affinity is strongly affected by transcription elongation blockage

**Figure 5:**
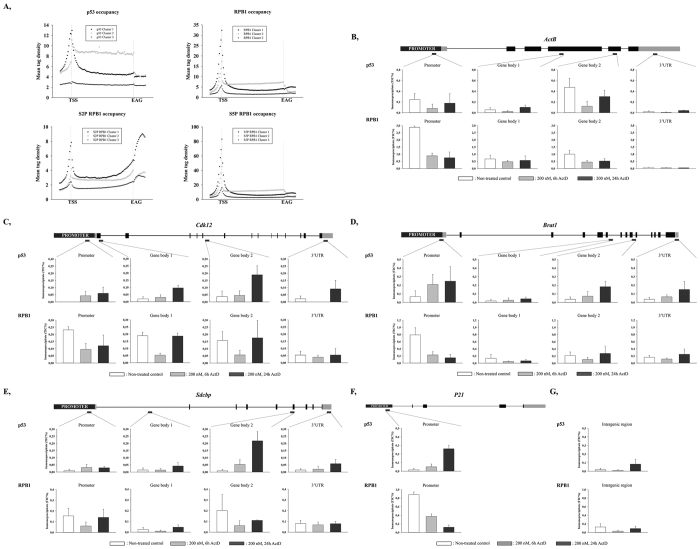
Human p53 binds to regions of genes which are not its direct targets. (**A**) ChIP-seq analysis of p53, RPB1, S2P RPB1 or S5P RPB1 occupancies results in the classification of 3 gene clusters. (**B**–**E**) p53 and RPB1 occupancies at the promoter, proximal (Gene body 1) and distal (Gene body 2) gene body and 3’UTR of *ActB, Cdk12, Brat1, Sdcbp* genes, respectively. On each graph, white columns represent untreated control, while grey and black columns indicate samples treated with 200 nM ActD for 6 and 24 h, respectively. The schematic structure for each gene and the location of the products amplified with qPCR are indicated. (**F**) For the positive control, p53 and RPB1 occupancies at the promoter of *P21*, a direct target gene of p53, are shown. (**G**) p53 and RPB1 occupancies at an intergenic region, which was used as a negative control. Each data point represents the average of measurements taken on two independent chromatin samples.

Since our data support the idea that p53 interacts with the elongating RNAPII complex, we re-analysed published ChIP-seq data that revealed the localisation of p53 (srr847010, srr847017), S2P RPB1 (srr987275), S5P RPB1 (srr987273) and RPB1 (srr987271) in U2OS cells, in order to validate whether p53 binds to transcribing units (Fig. 5A)^40^,^41^. We investigated p53 occupancy profiles on every gene annotated in RefSeq (https://www.ncbi.nlm.nih.gov/refseq) by calculating p53 tag densities ±1.5 kb distances around the annotated loci. By using a k-means clustering method on the occupancies of p53 and RPB1, we could sort the genes into three distinct clusters. Cluster 1 showed high p53 and RPB1 occupancies at the transcription start site. Cluster 2 genes showed elevated p53 and RPB1 bindings along the transcribed units. In Cluster 3 we could detect low RPB1 binding at the TSS (Transcription start site), while p53 could not be detected on those genes (Fig. 5A). These data suggest a correlation between p53 occupancy and gene expression level: p53 localises on the genes that show higher expression rate.

Since ChIP-seq data indicated that p53 was localised at specific genes and our data also revealed that p53 was associated with the elongating RNAPII, we decided to perform ChIP in order to validate the binding of p53 at specific gene regions (promoter, gene body and 3′UTR) of selected groups of genes which included *ActB* (Cluster 1), *Cdk12* (Cluster 1), *Brat1* (Cluster 1) and *Sdcbp* (Cluster 2). As a positive control, we examined the regulatory region of a well-known p53 regulated gene, *P21*, while as a negative control we included an intergenic region in our analysis.

We found that under normal conditions, p53 associated with specific regions of genes that are not its direct targets, as they do not contain canonical binding sites of p53. Under normal conditions, a low level of p53 occupancy could be detected at most of the examined gene regions (Fig. 5C–F, white columns). Only *ActB* seems to be an exception, since on this gene, a high level of p53 occupancy could be observed at the promoter and distal gene body (indicated as ‘Gene body 2’; Fig. 5B, white columns). RPB1 associated strongly at promoter regions and distal gene bodies of the examined genes, but showed weaker binding at the proximal gene bodies (indicated as ‘Gene body 1’) and at 3’ end regions of the genes (Fig. 5B–F, white columns).

To investigate whether p53 occupancy is altered by transcription elongation blockage, we measured the distribution of p53 and RPB1 at the examined gene regions using a similar experiment, but after treating cells with a high concentration (200 nM) of ActD for 6 and 24 h (Fig. 5B–G, grey and black columns, respectively). RPB1 occupancy was reduced 6 h after ActD treatment, and then it increased 24 h after transcription blockage induction, except at the promoter regions of *ActB, Brat1* and *P21* (Fig. 5B,D and F, respectively). Conversely, increased p53 binding was detected after ActD treatment, mainly at the promoters and distal gene bodies of the examined genes (*Cdk12, Brat1, Sdcbp* and *P21*), and this increase was time-dependent (Fig. 5C–F, respectively). In the case of the *ActB* gene (Fig. 5B), p53 occupancy showed a similar change over time as did RPB1: the binding level was reduced 6 h after ActD treatment (Fig. 5B, grey columns), then it was restored 24 h after transcription blockage induction (Fig. 5B, black columns).

These results show p53 accumulation at transcriptionally active chromatin regions upon transcription elongation blockage. Based on our data, we propose that p53 protein co-traverses with the elongating RNAPII and when transcription elongation blockage occurs, additional p53 protein molecules are recruited while the occupancy of RNAPII is reduced. We assume that p53 plays a role in the removal of the stalled RNAPII from the chromatin.

## Discussion

Here we report that p53 interacts with the transcriptionally competent forms of RNAPII and that p53 is present at transcriptionally active gene regions. Furthermore, we show that upon ActD-induced transcription blockage, the occupancy of RNAPII decreases, while p53 binding increases at regions of selected genes that harbour no canonical p53 sites. Our data suggest a novel role of p53, which might be required during transcription arrest.

Depending on its dosage, ActD can induce different molecular pathways. At a low concentration (5 nM), ActD mimics the effect of the Mdm2 inhibitor, nutlin-3, by abolishing RNAPI-mediated rRNA production. This leads to the accumulation of free ribosomal proteins, such as L11 and L23 that interact with and inhibit Mdm2. Conversely, at a high concentration (200 nM), ActD induces DNA double-strand breaks leading to the inhibition of transcription elongation^35^. Indeed, it has been demonstrated that ActD treatment—similarly to UV irradiation—can cause transcription elongation blockage by inhibiting the progression of the elongating RNAPII^42^,^43^.

During DNA double-strand break repair and UV-induced nucleotide excision repair, RNAPII becomes stalled at transcription units and these processes act as a so-called ‘last resort’ mechanism, finally activating the ubiquitylation and degradation of RNAPII^7^,^10^,^18^. UV-induced transcription blockage leads to p53 activation and helps the recruitment of DNA repair proteins to the DNA lesions, which can ensure genome stability and reduce the chance of tumour formation^8^. p53 activation by phosphorylation at its Ser-15 residue was also described as an essential step during DNA double-strand break repair. We found that transcription elongation blockage activated the Ser-15 phosphorylation of p53.

We detected the co-localisation of p53 with the S5P and S2P forms of RPB1. Interestingly, 24 h after treatment with a high concentration of ActD, p53 co-localised with RPB1 to discrete nuclear foci, presumably at the sites of DNA damage. This observation is in agreement with a previous demonstration that in *Caenorhabditis elegans*, upon transcription inhibition, RNAPII is localised to discrete chromatin “degradation centres” and is degraded by the 26S proteasome^21^. Another explanation for the co-localisation of S5P RNAPII and p53 could be that at these sites, transcription has already been reinitiated following the successful repair after ActD-induced DNA damage. Finally, these sites could mark stress-responsive genes, which remain transcriptionally active following DNA damage^44^.

Re-analysis of existing ChIP-seq data revealed that p53 and RNAPII recruited to promoters (Cluster 1) and gene bodies (Cluster 2) of highly transcribed genes during the normal transcription cycle. High p53 enrichment detected by ChIP-seq data was also confirmed by the chromatin immunoprecipitation method on *ActB, Cdk12, Brat1* and *Sdcbp* genes. Our data are in agreement with previous results^29^, which showed that in yeast, human p53 was bound to gene regions that do not contain any canonical p53 binding sites. At most of the examined regions of the four randomly chosen, non-direct p53 target genes, p53 occupancies increased after 6 and 24 h of transcription elongation blockage. On the other hand, RPB1 occupancy was reduced 6 h after ActD treatment and then restored after 24 h. *ActB*, however, behaved differently, since p53 and RPB1 occupancies showed similar distributions on this gene.

In this study, we showed that human p53 interacts with the largest subunit of RNAPII and that p53 co-traverses with the elongating RNAPII during transcription. If a transcription elongation blockage occurs, p53 and RPB1 show different distributions in their chromatin association: the binding of the p53 protein to chromatin increases, while RPB1 occupancy decreases. These results suggest that in human cells, p53 can regulate transcription elongation by a mechanism involving its interaction with RNAPII. Several studies have reported that the structural changes of the hyperphosphorylated S2P form of RPB1 promote its ubiquitin-mediated proteasomal degradation upon DNA damage^19^,^45^. In the case of UV-induced DNA damage, the hyperphosphorylation state of RPB1 does not allow a new PIC formation^44^,^46^. Furthermore, transcription blockage also induces the proteasomal degradation of RPB1^18^. Our results are in accordance with this phenomenon, as we report here that in human cells, upon induction of transcription elongation blockage, the degradation of the elongating form of RPB1 by the 26S proteasome might be influenced by p53.

Our data suggest that p53 may co-traverse with RNA polymerase II, similar to a transcription elongation factor, due to its interaction with RPB1. Our results indicate that RPB1 is degraded at the transcribed units by resolving the transcription blockage. This might serve to allow access for the repair proteins to the damaged DNA. The data we present in this paper provide a better understanding of how cells resolve transcription blockages when exposed to different stress conditions and the feedback mechanisms involved.

## Methods

### Cell lines, media and culture conditions

The U2OS cell line was cultured at 37 °C in DMEM (Dulbecco’s Modified Eagle Medium; Lonza) supplemented with 10% foetal calf serum (Lonza), 4 mM glutamine (Sigma-Aldrich) and 1x antibiotic (Sigma-Aldrich).

### Actinomycin D and MG132 treatment

Cells were treated with actinomycin D (ActD; Sigma-Aldrich) for 6 or 24 h. The cells were also treated with the proteasomal inhibitor, MG132 (Tocris Bioscience), 1 h before ActD treatment. ActD was used at concentrations of 5 nM or 200 nM and the final concentration of MG132 was 20 μM.

### Micrococcal endonuclease (MNase) treatment

We treated cells with MNase (Thermo Fisher Scientific) in order to study whether the interaction between p53 and the transcriptionally active forms of RPB1 was DNA-dependent. Before MNase treatment, 5 mM CaCl_2_ was added to each sample, which were then incubated for 1 minute at 37 °C. MNase was added to the samples at a concentration of 0.066 U/μl and they were incubated for 10 minutes at 37 °C. The samples were then placed on ice in order to inactivate the enzyme.

### ChIP-seq analysis

Published datasets were downloaded from The European Nucleotide Archive (http://www.ebi.ac.uk/ena) under the following accession numbers: p53 and related input files (srr847010, srr847017); S2P RPB1 (srr987275); S5P RPB1 (srr987273); RPB1 (srr987271). All data were generated from the U2OS cell line.

The reference human genome (GRCh38/hg38 assembly) and annotated gene list were downloaded from the UCSC Genome Browser (www.ucsc.org). For the analysis described here, we have considered only the RefSeq genes that are reviewed and validated.

ChIP-seq tag density values were extracted for every RefSeq gene over the annotated gene body and + /−1500 bp upstream and downstream from the loci, using the program seqMINER^43^,^47^. The average gene profile of ChIP-seq tags and differential binding patterns were identified using the methods described in Anamika *et al*.^44^,^48^.

### Chromatin immunoprecipitation

Chromatin samples were prepared from U2OS cells. Cells were cross-linked with 1% formaldehyde (Sigma-Aldrich) for 10 minutes then incubated with 125 mM glycine (Sigma-Aldrich) for 5 minutes to stop fixation. Cells were collected using centrifugation for 5 minutes at 2000 rpm and 4 °C. Cell pellets were resuspended in a cell lysis buffer [5 mM PIPES (Sigma-Aldrich) pH 8.0, 85 mM KCl (Sigma-Aldrich), 0.5% NP-40 (IGEPAL; Sigma-Aldrich), 1x PIC-C (Protease Inhibitor Cocktail, Calbiochem)] and incubated on ice for 10 minutes. Cells were collected using centrifugation (2000 rpm, 5 minutes, 4 °C). Pellets were resuspended in a nuclear lysis buffer [50 mM Tris-HCl pH 8.0 (Sigma-Aldrich), 10 mM EDTA pH 8.0 (Sigma-Aldrich), 0.8% SDS (Sigma-Aldrich), 1x PIC-C (Calbiochem)] and incubated on ice for 1 h. Chromatin samples were fragmented by sonication using Bioruptor (Diagenode). Chromatin samples were diluted four-fold with a dilution buffer [10 mM Tris-HCl pH 8.0 (Sigma-Aldrich), 0.5 mM EGTA pH 8.0 (Sigma-Aldrich), 1% Triton-X-100 (Sigma-Aldrich), 140 mM NaCl (Sigma-Aldrich), 1x PIC-C (Calbiochem)]. 30 μg of pre-cleared chromatin was used for each immunoprecipitation. Immunoprecipitations were incubated overnight at 4 °C with the antibodies listed below, then chromatin-antibody complexes were collected with 40 μl Sheep anti-Rabbit IgG Dynabeads (Novex) at 4 °C. After washing, chromatin-antibody complexes were eluted from beads, then DNA fragments were precipitated overnight at −80 °C. After centrifugation, pellets were resuspended in TE (Tris-EDTA) buffer then reverse cross-linked at 50 °C for 2 h. DNAs were purified with phenol-chloroform extraction, then precipitated overnight with absolute ethanol at −80 °C. The amount of extracted DNA was determined by Q-PCR using SYBR Green PCR Master Mix (Fermentas) in Thermo PikoReal 96 Real-Time PCR system (Thermo Fisher Scientific). Sequences of primers used in the Q-PCR are listed in Supplementary Table S1.

Samples were quantified using a TIC (total input control) standard curve. The amount of DNA specifically precipitated by the given antibody was calculated by deducting the amount of DNA in the no-antibody control (NAC) from the total. The following antibodies were used in the ChIP experiments: hp53 ab17990 (Abcam), RPB1 (N-20) and sc-899 (Santa Cruz).

### Co-immunoprecipitation

U2OS cells were lysed in a lysis buffer (150 mM NaCl, 1% Triton X-100, 50 mM Tris-HCl pH 8.0) containing 1x PIC-C (Calbiochem) and 1X PhosSTOP (Roche) on ice for an hour. After lysis, cell debris was pelleted by centrifugation at 2000 rpm, 4 °C for 5 minutes, then was discarded. 300 μg lysates were pre-cleared for 2 h with blocked Protein A-Sepharose beads (Sigma-Aldrich). Polyclonal RNAPII antibody against S5P or S2P CTD (Abcam) was used for the immunoprecipitation. To control the co-immunoprecipitation, we used a p53 (Abcam) antibody for the reverse experiment. For detection of non-specific protein binding (IgG control) we added Protein A-Sepharose beads to the cell lysate, without a specific antibody. The protein-antibody complexes were collected with 40 μl of blocked Protein A-Sepharose beads (Sigma-Aldrich). Then beads were washed for four times with lysis buffer supplemented with 1x PIC-C (Calbiochem). After washing, beads were boiled in 2x SDS loading buffer for 5 minutes and centrifuged at 13,000 rpm for 5 minutes at 4 °C. To detect the interaction between RPB1 and the p53 protein, monoclonal hp53 antibody (Dako) was used in 1:750 or 1:1500 dilutions and polyclonal S15P p53 antibody (Cell signalling) was used in 1:250 or 1:500 dilutions in the immunoprecipitated and input samples, respectively. For reverse immunoprecipitation, the antibody against S5P CTD of RPB1 (Abcam) was used in 1:1000 or 1:2000 dilutions, and the antibody against S2P CTD of RPB1 (Abcam) was used in 1:500 or 1:1000 dilutions, in the immunoprecipitated and input samples, respectively. For p53 detection, we used the anti-kappa light chain secondary antibody (from L. Tora, IGBMC).

### Immunocytochemistry

U2OS cells were washed with PBS (Phosphate-buffered saline), then rinsed twice for 3 minutes with CSK (Cytoskeletal) buffer [10 mM HEPES pH 7.0 (Sigma-Aldrich), 100 mM NaCl (Sigma-Aldrich), 300 mM sucrose (Molar Chemicals), 3 mM MgCl_2_ (Molar Chemicals), 0.7% Triton-X-100 (Fluka) and 0.3 mg/ml RNase A (Sigma-Aldrich)]. Cells were rinsed twice with PBS, then fixed with 4% formaldehyde (Sigma-Aldrich) for 10 minutes. After washing, cells were permeabilised for 5 minutes in PBS containing 0.2% Triton-X-100 (Fluka). Non-specific staining was blocked with 5% BSA in PBST [0.1% Tween 20 (Molar Chemicals) in PBS] for 20 minutes. Cells were incubated with the following primary antibodies: anti-p53 (Dako, IS616), N-20 anti-RPB1 (Santa Cruz, sc899), anti-S2P RPB1 (Abcam, ab5095), anti-S5P RPB1 (Abcam, ab5131), anti-S139P H2AX (Millipore, 05-636-I) and anti-S139P H2AX (ab2893). After washing, the following secondary antibodies were used: GAM Alexa 488 (Molecular Probes, A11029) and GAR DyLight 550 (Abcam, ab96984). Both the primary and secondary antibodies were applied in 1% BSA-PBST. Coverslips were mounted on glass slides using DAPI containing ProLong Gold Antifade reagent (Life Technologies). Samples were visualised with Olympus FluoView FV1000 confocal microscope. The same exposition time was used to capture each image.

### Western blot

U2OS cells were harvested in lysis buffer (150 mM NaCl, 1% Triton X-100, 50 mM Tris-HCl pH 8.0; Sigma-Aldrich) supplemented with 1x PIC-C (Calbiochem), incubated on ice for an hour, then centrifuged (13,000 rpm at 4 °C for 5 minutes). The supernatant lysates were mixed with the same amount of 2x SDS loading buffer containing 5% β-mercaptoethanol (Sigma-Aldrich) and boiled for 5 minutes. The lysates were separated in SDS-PAGE, transferred to Amersham Hybond ECL-membrane (GE Healthcare) and incubated with the following primary antibodies: anti-p53 (Dako, IS616), anti-S15 P p53 (Cell signalling, 9284), 1BP7G5 anti-RPB1 (from L. Tora, IGBMC), anti-S2P RPB1 (Abcam, ab5095) and anti-S5P RPB1 (Abcam, ab5131), anti-GAPDH (Millipore, MAB374); then the following secondary antibodies: RAM-HRP (Dako, P0260) and GAR-HRP (Dako, P0448). Chemiluminescent detection was conducted using Immobilon Western Chemiluminescent HRP substrate (Millipore).

## Additional Information

**How to cite this article**: Borsos, B. N. *et al*. Human p53 interacts with the elongating RNAPII complex and is required for the release of actinomycin D induced transcription blockage. *Sci. Rep.* **7**, 40960; doi: 10.1038/srep40960 (2017).

**Publisher's note:** Springer Nature remains neutral with regard to jurisdictional claims in published maps and institutional affiliations.

## Supplementary Material

Supplementary Figures and Table 1
