# Supplementary material for: Human p53 interacts with the elongating RNAPII complex and is required for the release of actinomycin D induced transcription blockage
Source: Sci Rep. 2017 Jan 19;7:40960. doi: 10.1038/srep40960 (PMC5244413; doi:10.1038/srep40960)
Supplement: Supplementary Figures and Table 1 [file srep40960-s1.pdf]

Human p53 interacts with the elongating RNAPII complex and is required for the release of actinomycin D induced transcription blockage

Barbara N. Borsos, Ildikó Huliák, Hajnalka Majoros, Zsuzsanna Újfaludi, Ákos Gyenis, Peter Pukler, Imre M. Boros, Tibor Pankotai

Supplementary informations:

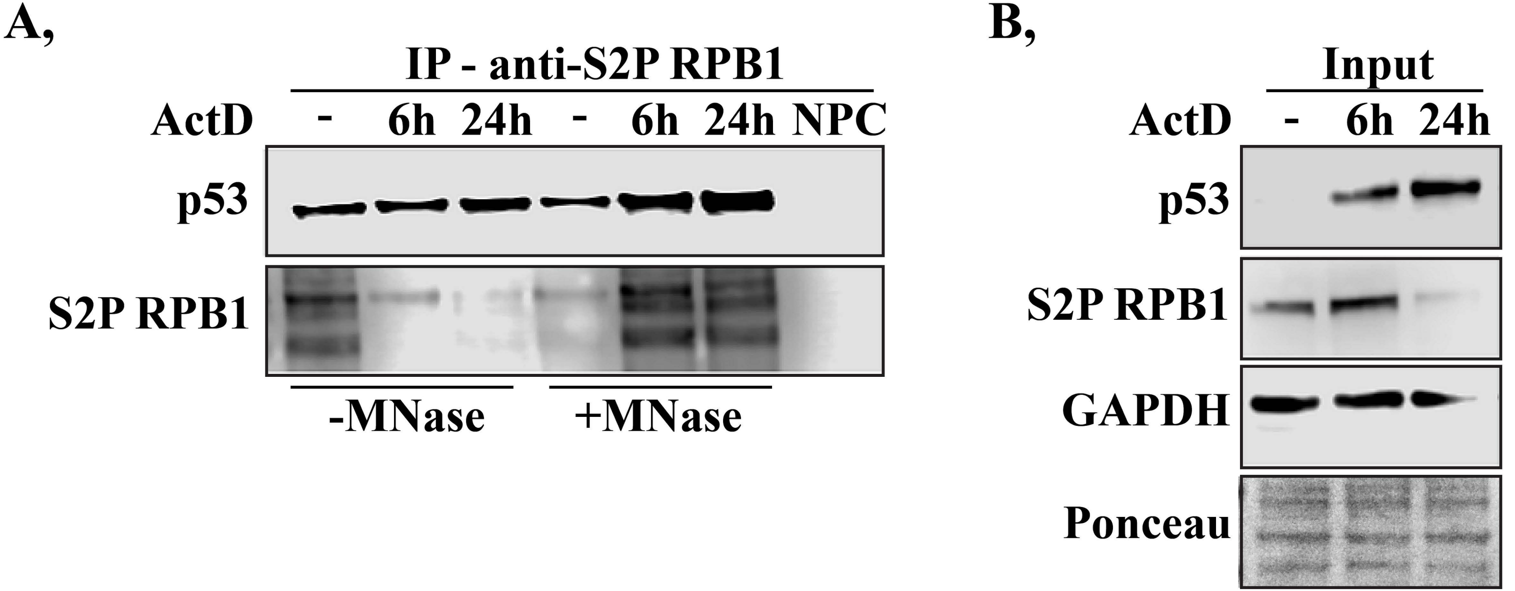

Supplementary Figure S1. Co-immunoprecipitation of p53 and S2P RPB1

(A) S2P RPB1 was co-immunoprecipitated with p53. IP was performed by anti-S2P RPB1 antibody and anti-p53 was used to detect p53 in the precipitated samples. „No protein control" (NPC) was used as negative control of the immunoprecipitation. In each case, MNase treatment was performed both under normal and ActD treated (200 nM, 6 and 24 h) conditions. (B) Western blot detection of p53 and S2P RPB1 in the inputs of immunoprecipitaion. GAPDH was used to show the equal loading.

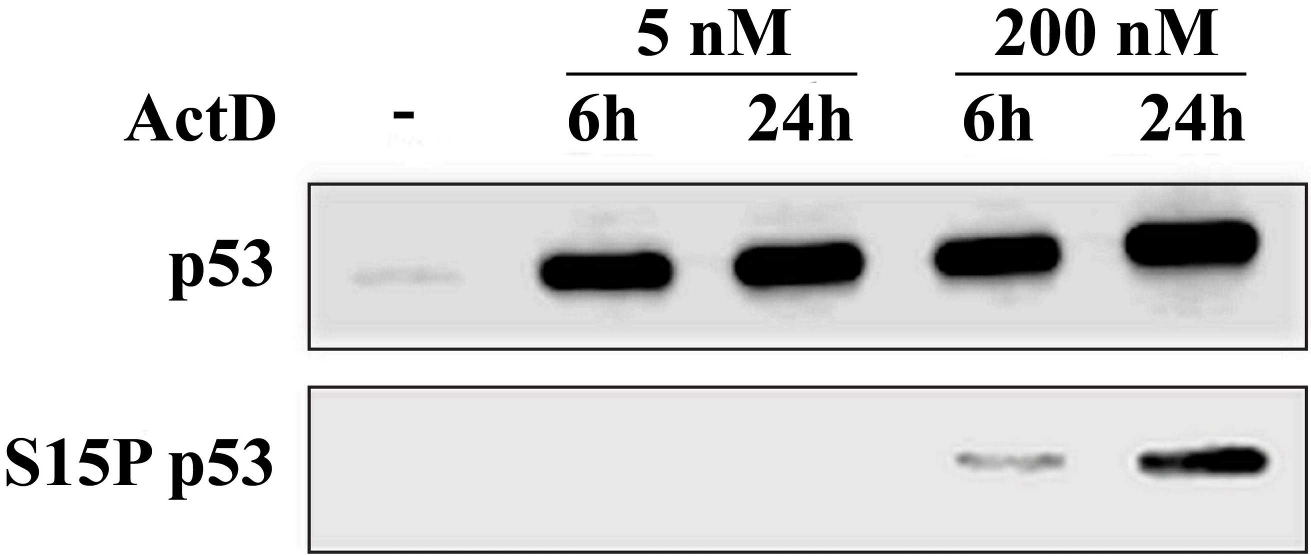

Supplementary Figure S2 p53 protein is stabilized upon ActD treatment
Western blot detection of p53 and S15P p53 6 and 24 h after 5 and 200 nM ActD treatment.

Gene region	Forward primer (5'-3')	Reverse primer (5'-3')
ActB promoter	CGCCAAAACTCTCCCTCCTC	CCGCTGGGTTTTATAGGGCG
ActB gene body 1	CTTTCTCTGCACAGGAGCCT	AGGTGTGGTGCCAGATTTTC
ActB gene body 2	GTGAGGACCCTGGATGTGAC	GCAAAGACCTGTACGCCAAC
ActB 3'UTR	CACACAGGGGAGGTGATAGC	ACATCTCAAGTTGGGGGACA
Cdk12 promoter	GGAGCCTGGGAGTGTTTGTT	CGCTTTCACCTCATTCCCCT
Cdk12 gene body 1	CCCCAGGTGAGCTATTTGTC	CAACTGAAGACCCCACCACT
Cdk12 gene body 2	GCAGTGAGCCGAGATTGAGT	GCCTGGAGGTTGAGGATGTA
Cdk12 3'UTR	ACATCCCAGCATGTGTACCC	CCTCCAAATTATCCCATCC
Brat1 promoter	GTGGGGAACTGAGACTGCAC	CTCGGCATGAACCACTAGG
Brat1 gene body 1	CTCCTAATGTCCCCAGCAAG	AGGCCCCTCAGTATCTGCTC
Brat1 gene body 2	CGAGCCAGCTACTCTCATCC	ACTCACCTCGCGTTTGTCTC
Brat1 3'UTR	CTGTGGGATTTCTTGACCTTG	CAGCACCATGTCCTTCTGTG
Sdcbp promoter	AGGAGTTGAGAAGGGGTCGT	GTCGCCTCCAATTCAAAGAG
Sdcbp gene body 1	TTTGCCAGTGTTTCCTACTTCA	TTTCCCATTTGTCCCTCAAC
Sdcbp gene body 2	TGGTTTTGTCAGCAGTTGGT	GCCTGAGTCCAATTTTTCCA
Sdcbp 3'UTR	CCCCTTTTGTTCATTTGTGG	GCAAGGAGGAGTCAGGAAAA
P21 promoter	CCGAAGTCAGTTCCTTGTGG	CGCTCTCTCACCTCCTCTGA
Intergenic region	TGGAACTTCTGGAAGACACTG	TACACCACTCAAGGGAAACTG

Supplementary table S1. Sequences of primers used in chromatin immunoprecipitation experiments.
